# Flushing as a Control Measure for *Legionella* spp.: Impacts of Water Age, Chloramine Disinfection, and Elevated Temperature

**DOI:** 10.1021/acs.est.5c16399

**Published:** 2026-01-28

**Authors:** Charuka S. Meegoda, Michael B. Waak, Taegyu Kim, Raymond M. Hozalski, Cynthia Hallé

**Affiliations:** † Department of Civil and Environmental Engineering, Norwegian University of Science and Technology, 7031 Trondheim, Norway; ‡ Department of Infrastructure, 87537 SINTEF Community, 7031 Trondheim, Norway; § Department of Civil, Environmental, and Geo-Engineering, 5635 University of Minnesota, Minneapolis, Minnesota 55455, United States

**Keywords:** flushing, premise plumbing, chloramine, biofilm, water age

## Abstract

Pilot-scale experiments were used to examine how hot-water temperature, chloramine disinfection, and pipe material (copper or PE-Xa) influence the effectiveness of two flow-based interventions—high-flow flushing vs showering—in controlling total bacteria, *Legionella* spp., and *Vermamoeba vermiformis* in premise plumbing. Flushing (ca. 16 L/min for 5 min) and showering (ca. 7 L/min for 8 min) both temporarily reduced bacterial concentrations by removing accumulated planktonic biomass. However, total bacteria rebounded within 1–4 days after a flush or shower, regardless of the presence of chloramines (1 mg/L Cl_2_). In the absence of chloramines, *Legionella* spp. *ssrA* gene concentrations increased over 3–10 days following flushing or showering, whereas chloramines suppressed *Legionella* spp. for >10 days despite completely decaying within hours of water use. Without chloramines, hot-water flushing at 49 or 60 °C provided little additional control of *Legionella* spp. as compared to cold-water flushing. *V. vermiformis* concentrations decreased temporarily following water use, independent of disinfectant or temperature. Flushing was more effective than showering at removing *Legionella* spp. from biofilms, while pipe material had limited influence in this pilot-scale system. Overall, the results demonstrate organism-specific recovery dynamics and highlight the role of chloramines in prolonging the suppression of *Legionella* in premise plumbing.

## Graphical abstract

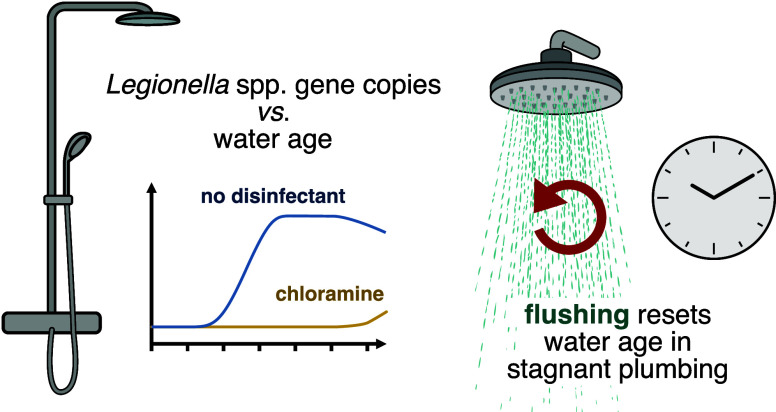

## 1 Introduction

*Legionella pneumophila*, along with multiple other *Legionella* spp., are opportunistic pathogens capable of causing Legionnaires’ disease and Pontiac fever.^1^ Although Legionnaires’ disease accounts for only 5% of community-acquired pneumonia cases, it is among the top three requiring intensive care unit admission and has mortality rates between 4 and 40%.^2,3^ Disease incidence has been increasing in many countries globally.^4,5^ This trend gained heightened awareness and concern during the COVID-19 pandemic due to decreased occupancy of many nonresidential buildings and the resulting stagnation in the water systems of those buildings.^6–9^

*Legionella* spp. are commonly found in natural and engineered aquatic environments, including municipal drinking water networks and building water systems.^10–13^ Considered slow-growing, *Legionella* multiply at temperatures ranging from 20–50 °C, with optimal growth typically reported around 40 °C but varying by strain.^14^ Their growth in water is facilitated by parasitizing amoebic or ciliate hosts or by residing in biofilms.^15^ Although *Legionella* are sensitive to temperatures above 55 °C and common disinfectants like free chlorine (HOCl) and monochloramine (NH_2_Cl), association with protozoa or biofilms can enhance their survival in hostile conditions.^16–20^ Thus, in building water systems, factors such as long residence times, warm temperatures, reduced disinfectant concentrations, and high surface area to volume ratios can promote the proliferation and persistence of *Legionella*, affecting occupant exposure risk.^21,22^ Human exposure typically occurs via inhalation of contaminated aerosols, with showers, faucets, and toilets representing potential aerosol sources.^23^

Flushing is a widely recommended strategy for limiting *Legionella* colonization, growth, and transmission in building water systems.^24–29^ Guidance documents, however, often define flushing inconsistently, with recommended duration, frequency, outlet type, and temperature (or blending) varying—or not specified at all. Recent works have also suggested that flushing yields limited benefits in mitigating *Legionella* contamination.^8,30^ We previously demonstrated that flushing shower outlets with water lacking disinfectant temporarily decreased accumulated *Legionella* spp. concentrations, though elevated levels re-emerged within days.^31^ Hozalski et al.^6^ likewise reported that flushing with chloraminated municipal drinking water for as little as 6 min could eliminate *Legionella* spp., but their presence was observed again after only 2 or 3 days of stagnation (i.e., increased water age due to lack of water use). Given the costs of flushing in terms of water and energy usage as well as personnel time, evidence-based protocols are needed to maximize flushing benefits relative to cost and likewise clarify the key mechanisms contributing to flushing success.

In this study, we assessed the immediate and long-term effects of flushing at maximized water flow rates on both total bacteria and *Legionella* spp. in water and biofilm phases by performing controlled experiments using a pilot-scale building water system. Flushing was performed with cold water (ca. 10 °C) and with hot water at either 49 or 60 °C in the presence and absence of residual chloramines. Additionally, we compared the effect of copper versus cross-linked polyethylene (PE-Xa) pipe material and the benefits of flushing at maximized flow rates (i.e., by removing the shower hose and head) versus conventional water use via showering.

## 2 Materials and Methods

### 2.1 Pilot-Scale Building Water System

Meegoda et al.^31^ and Waak et al.^32^ describe a pilot-scale building water system in Trondheim, Norway, used to study the effects of flushing on suspended biomass in hot and cold water and attached biomass in pipe biofilms (Figure 1). In summary, the system included two parallel rigs fed with municipal tap water via the laboratory plumbing and a check-valve to prevent backflow. Copper pipes with compression fittings supplied both rigs with cold water, including a water heater and three trellis-mounted shower mixers per rig. The distal 1 m of cold- and hot-water piping to each shower mixer was removable to facilitate biofilm sampling. These distal pipes were comprised of copper in one rig and Uponor Aqua PE-Xa in the other, with internal diameters of 16.0 and 12.5 mm, respectively.

**Figure 1:**
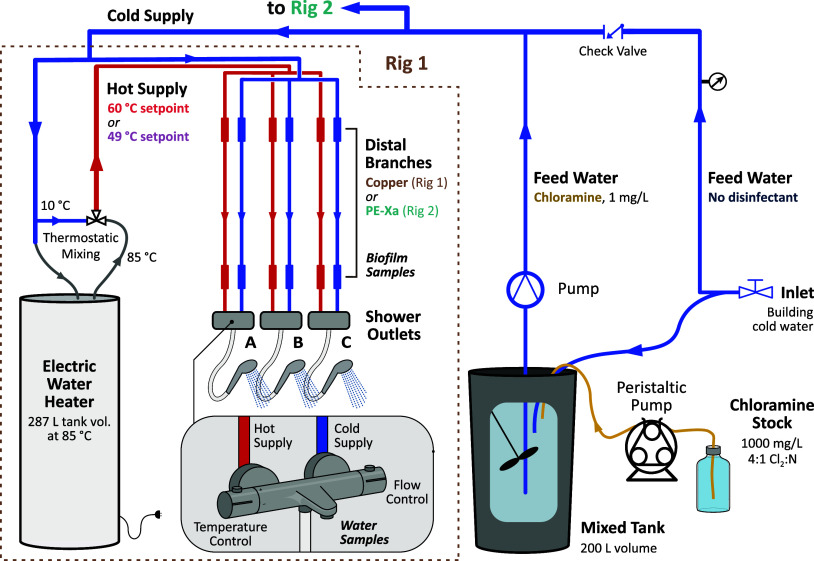
Conceptual schematic of the pilot-scale building water system, which consisted of two independent rigs in parallel. Adapted with permission from Waak et al.^32^ Copyright 2024 Water Research Foundation.

Hot water for each rig was supplied by a separate 2.8 kW electric water heater (OSO Hotwater, 287 L SAGA S 300). Heated water (85 °C) was blended with cold tap water (ca. 10 °C) at the heater using a thermostatic mixing valve (ESBE VTA321) to deliver hot water to the rig piping at either 49 °C (1:1 mixing; ca. 500 L capacity) or 60 °C (7:3 hot/cold mixing; ca. 400 L capacity). Residential water heaters in Norway are commonly factory preset at 70–75 °C for *Legionella* prevention, with thermostatic mixing used to reduce temperatures prior to the points of use for scald prevention. In this study, the heater thermostat was set to 85 °C solely to increase hot-water production capacity during repeated flushing and showering. Delivered hot-water temperatures within the rig piping were controlled exclusively by the thermostatic mixing valves, and the heater set point remained constant across all experimental phases and was not an experimental variable.

For chloramination during pilot operation, building tap water was bypassed into a 200 L mixed tank fed by a Cole-Parmer MasterFlex L/S peristaltic pump with 1000 mg/L chloramine stock solution (primarily NH_2_Cl) to a target, steady-state concentration of 1 mg/L Cl_2_ (total chlorine). The stock was prepared as described by Alexander et al.^33^ (Cl_2_:N = 4:1, pH 11.1 ± 0.3) and stored at 4 °C for up to 3 days when not in use. A submersible pump (250 W) was suspended in the mixed tank to provide up to 130 L/min nonvortex mixing of the continuous feeds of tap water and chloramine stock, with excess chloraminated water directed by overflow to the building sewer system. The chloraminated water was supplied to the rigs by a Grundfos 550 W SCALA2 3–45 A water booster pump.

### 2.2 Municipal Tap Water Quality

The Trondheim municipal drinking water supply is distributed without a persistent disinfectant residual. In the primary water source, lake water is withdrawn at 50 m depth and then passed through granular marble beds to increase alkalinity and water hardness for corrosion control. Water is disinfected with variable-dose free chlorine (about 0.2 mg/L Cl_2_) for a 30 min contact time, followed by UV irradiation with medium-pressure lamps (40 mJ/cm^2^). Finished water contains 0.08 ± 0.01 mg/L Cl_2_ free chlorine. Water quality is summarized in Table S1 in the Supporting Information.

### 2.3 Pilot Operation and Experimental Design

The pilot-scale building water system was operated for four months to inoculate the pipes with the native drinking-water microorganisms and stabilize the new copper pipes from corrosion. The system was then operated for approximately one year, February 2021 to March 2022, to assess different experimental variables (Table S2) within a factorial study design (Figure S1). Briefly, the effects of disinfectant residual and hot-water temperature were assessed with 6- to 10-week periods of operation under each combination of variables: 49 or 60 °C hot water, with or without residual chloramines. For replication, three water-usage cycles were conducted during each period of operation, consisting of 10–21 days of stagnation in all shower outlets followed by 4 days of active water use (i.e., once-daily simulated showers) in two of the three outlets per rig (Figure S2). In both pilot rigs, the transition from low to active water usage was marked by flushing one shower outlet (“flushed”) and operating another outlet with a simulated shower without flushing (“no flush”). The third outlet (“stagnant”) in each rig was kept stagnant to provide an intact biofilm sample without exposure to flow. The role of each outlet was shuffled after each set of three flushing cycles, and there were also transition phases of at least 2 weeks when hot-water temperature changed and 7 weeks when chloramination started, to foster microbiome acclimation to the new conditions.

Flushing was performed by disconnecting a shower hose from its outlet, setting the temperature mixer control to full cold or hot, and then turning the flow control to its maximum for 5 min (15.7–17.5 L/min). In contrast, simulated showers were 40 °C and lasted for 8 min with a target flow rate of 7.2 L/min, based on the average duration and flow rate for showers in the United States.^34^ Flow rates were determined by dividing the total volume of water by the flush or shower duration. The flow velocity, Reynolds number, and shear stress in the distal branches during flushing and showering were calculated as previously described^32^ and summarized in Table S3.

### 2.4 Chloramine Decay Experiment

In situ chloramine concentrations in the pilot system were monitored over a 2-day period to compare decay rates with those observed by Hozalski et al.^6^ in full-scale buildings. We also measured chloramine decay rates in glass bottles as controls. Chloraminated water samples (1 L) were collected from the pilot cold and hot supplies after simulating a shower. Samples were collected in glass bottles, prepared by washing with 0.2 M HCl, rinsing with ultrapure water (Milli-Q), and baking at 500 °C for 1 h. Finally, another control bottle was prepared by dosing ultrapure water with 1 mg/L chloramine. The bottles were incubated in the dark at room temperature (ca. 20 °C) for 1 month with periodic measurement of total chlorine levels. The first-order chlorine decay rate constants were determined in R with the model by Clark et al.^35^

### 2.5 Water Samples

All water samples were collected in sterile Nasco Whirl-Pak Stand-Up Bags. Before flushing, 150 mL samples of stagnant water were taken from the hot and cold supplies of each shower outlet. These samples served as a preflush reference and assessed the impact of water age (10+ days) on water quality from the previous cycle. After flushing, 1.1 L of fresh hot and cold water was collected from the flushed outlets.

Additional water samples were collected from shower outlets at 2 and 7 days after flushing. On day 2, 150 mL of stagnant cold and hot water (aged 1 day) were collected before showers from all outlets, and 1.1 L of fresh cold and hot water was collected after showers from the previously flushed outlet only. On day 7, only stagnant water (150 mL) was collected from each hot and cold pipe, with an approximate water age of 3 days, as daily shower use was stopped on day 4. Extended water ages up to 28 days were assessed by sampling the stagnant water just prior to the next flushing cycle.

### 2.6 Water Quality Analysis, Flow Cytometry, and Filtration

Immediately after collection, aliquots of the water samples were tested for temperature, pH (Xylem SenTix 81), turbidity (Hach 2100Q), conductivity (VWR CO 3000L), and total chlorine (Hach DPD method). Total organic carbon (TOC) was measured as nonpurgeable organic carbon (NPOC) using a Teledyne-Tekmar Apollo 9000 analyzer following Standard Method 5310B (high-temperature combustion); samples were acidified with phosphoric acid prior to analysis to remove inorganic carbon. Instrument calibration and quality control were verified using method blanks and standards.

The remaining water was either analyzed immediately or stored up to 8 h at 4 °C until analysis. Sodium thiosulfate was added to chloraminated water upon collection to quench the residual disinfectant (0.1 mL of 3% Na_2_S_2_O_3_ per 100 mL sample). About 5 mL of water was analyzed using a BactoSense Online Flow Cytometer with an ICC-type cartridge (bNovate), where total and intact cell counts were determined from 90 μL of sample following a universal gating strategy (Figure S3).^32,36^

To isolate biomass from water for DNA extraction, 100 or 1000 mL of sample was vacuum-filtered via 0.22 μm, 47 mm mixed cellulose membranes (MilliporeSigma). Negative controls were prepared by vacuum-filtering 1 L of autoclaved ultrapure water (Millipore Milli-Q). The filters were cut aseptically into 5 mm squares, added to ZR BashingBead Lysis tubes with 1.0 mL DNA/RNA Shield (Zymo Research), and stored at –20 °C until extraction.

### 2.7 Biofilm Samples

On flushing days, biofilm samples were collected approximately 4–5 h after water samples. During this process, the water to the pilot was shut off, and the distal hot and cold branches were disconnected from each shower outlet by unscrewing the lower fitting and pulling the branch forward, while the top of the branch remained connected to the pilot. For the purposes of sampling, steel wires were formed into loops using cable clamps. To sample, a sterile POLYWIPE sponge (Medical Wire & Equipment Co.) was fastened in a wire loop secured in a cordless electric drill (Bosch EasyDrill 18 V-40). The drill was operated in gear 1 (manufacturer-rated speed range: 0–430 rpm) at approximately midrange trigger position, corresponding to an estimated rotational speed of ca. 215 rpm. The loop/sponge assembly was inserted into the pipe and spun for 30 s clockwise and 30 s counterclockwise. The sponge was transferred to a Nasco Whirl-Pak bag containing 50 mL of autoclave-sterilized elution solution^37^ (100 mg/L (NaPO_3_)_*n*_, 0.01% Tween 80, and 0.001% antifoam Y-30 emulsion). This was sonicated for 2 min using an ultrasonic bath to suspend biofilm from the sponge (Elmasonic S 15 H, Elma Schmidbauer GmbH, Germany; 37 kHz, effective ultrasonic power 35 W). The elution solution was then pressed out of the sponge and transferred to a second Whirl-Pak bag. Another 50 mL of elution solution was added, hand-squeezed with the sponge, and combined with the first suspension for approximately 100 mL total. Finally, the suspension was vacuum-filtered through a membrane and stored at −20 °C in DNA/RNA Shield, identical to water samples. Negative controls were prepared using sterile POLYWIPE sponges without biofilm contact.

### 2.8 DNA Extraction

Preserved filter membranes were thawed and homogenized using a Bertin Instruments Precellys Evolution with the Zymo Research lysis protocol: 1 min at 9000 rpm, four times with 2 min rest intervals. DNA was extracted using the ZymoBIOMICS DNA Miniprep Kit (Zymo Research) and stored in 100 μL nuclease-free water at −20 °C.

### 2.9 Real-Time qPCR

Various taxa were quantified by real-time quantitative polymerase chain reaction (qPCR) on a Bio-Rad CFX Connect Real-Time PCR Detection System: total bacteria using the 341*F*/534R primers^38^ of bacterial 16S rRNA genes, *Legionella* spp. targeting *ssrA* using PanLegF and PanLegR primers with probe PanLegP,^39^ *L. pneumophila* targeting its *mip* gene with primers LpF/LpR and probe LpP,^39^ and the amoeba host, *Vermamoeba vermiformis*, using primers Hv1227F/Hv1728R^40^ targeting its 18S rRNA genes. All primers and probe PanLegP were synthesized by Integrated DNA Technologies; LpP was a TaqMan probe (ThermoFisher Scientific).

Reactions consisted of 10.0 μL of either Bio-Rad SsoFast EvaGreen Supermix with Low ROX (bacterial 16S rRNA genes and *V. vermiformis* 18S rRNA genes) or Bio-Rad SsoAdvanced Universal Probes Supermix (*ssrA* and *mip*), 20 μg bovine serum albumin (Roche Diagnostics), 2.0 μL DNA extract, and then primers and probe plus PCR-grade water (Sigma-Aldrich) for 20.0 μL final volume. PCR conditions, primer/probe sequences and their final concentrations are summarized in Table S4.

Reference genes for each of the genetic targets (Table S5) were synthesized as gBlocks gene fragments (Integrated DNA Technologies). These were serially diluted to produce calibration curves (Table S6). For 16S rRNA genes, a limit of quantification (LOQ) was defined as 1970 copies per reaction using the negative controls: the mean gene copy number plus ten times the standard deviation. For *ssrA*, *mip*, and 18S rRNA genes, the LOQ was operationally defined as the lowest calibration standard (10 copies per reaction).

### 2.10 Statistical Analysis, Hypothesis Testing, and Modeling

Analytical data were manually quality-controlled and analyzed in R software^41^ using the *stats*, *emmeans*, *car*, *marginaleffects*, *NADA2*, and *tidyverse* packages.^42–46^ Hypothesis testing followed predefined research questions that focused on (i) quantifying the immediate effects of outlet operation (flushing or showering) on microbial concentrations, (ii) comparing flushing versus showering as alternative flow-based interventions, and (iii) assessing whether disinfectant residual, pipe material, or hot-water temperature modified these immediate effects.

Immediate effects were quantified as within-event changes in log_10_-transformed qPCR gene target concentrations (post- minus prewater use). This differencing removed event-specific baseline conditions, rendering the analysis analogous to paired comparisons and avoiding the need for random intercepts. These log_10_ reductions were analyzed using linear models with experimental factors treated as fixed effects. Cold and hot water data were modeled separately, with temperature set-point included only for hot water. Linear model specification and selection is further described in the Supporting Text.

Model selection was based on the Akaike information criterion (AIC) and was used to evaluate whether experimental factors acted additively or through interaction effects. Competing models with additive effects were compared against models including higher-order interactions among intervention, disinfectant, pipe material, and temperature. In all cases, additive models were favored over interaction models, indicating no statistical support for interaction effects under the conditions tested. Statistical significance was evaluated at α = 0.05.

Main effects were examined using post hoc pairwise contrasts with multiplicity adjustment according to Holm.^47^ Marginal means, contrasts, and average marginal effects (slopes) were estimated to facilitate interpretation of effect sizes on the log_10_ reduction scale.

Quantitative qPCR measurements below the method quantification limit, including reactions with no detectable amplification, were flagged as left-censored during data processing. For water-phase analyses, immediate effects were evaluated using log_10_ concentration changes. Observed qPCR values were retained even when below the method quantification limit, and substitution at the quantification limit was applied only in cases of nondetects (i.e., no amplification), which did not occur for 16S rRNA gene or *ssrA* targets in water samples. Biofilm-associated qPCR data exhibited substantial left-censoring and violated the assumptions required for linear modeling. Accordingly, biofilm responses to flushing and showering were evaluated using nonparametric paired Wilcoxon signed-rank tests for censored data, with multiplicity adjustments using the Holm method.^47^

Bacterial growth dynamics during postflush or postshower stagnation were modeled in R using the *nlsMicrobio*, *minpack.lm*, *gslnls*, *stats*, and *broom* packages.^41,48–51^ Growth parameters^52^—including the initial concentration (*N*_0_), lag phase duration (λ), maximum specific growth rate (μ_max_), doubling time (τ), and stationary-phase concentration (*N*_max_)—were estimated by fitting log_10_-transformed qPCR-derived gene target concentrations to water age. Both linear and nonlinear models were evaluated, including variants of the Baranyi and Roberts model^53^ and simple linear models,^54^ with final model selection based on the lowest AIC.^32^

## 3 Results

### 3.1 Chemical Water Quality Indicators

The temperature of the cold water varied over the course of the experiments with the lowest temperature during spring testing (mean ± standard deviation; 7.0 ± 0.9 °C) and the highest during fall testing (13.1 ± 0.3 °C). The mean hot water temperatures were 59.2 ± 1.0 °C and 47.2 ± 0.9 °C when the target values were 60 and 49 °C, respectively. During a simulated daily shower, the blended cold and hot water temperature stabilized within 2 min of operation at 40.6 ± 0.3 °C (target = 40 °C). The temperature of the water in the lines returned to room temperature (22.2 ± 0.8 °C) after a few hours of postshower or postflush stagnation.

The pH was consistently around 8 throughout all phases of testing. The mean pH of the flowing water after 8 min of shower operation (8.02 ± 0.15) was unchanged from that after periods of stagnation (7.99 ± 0.30). However, the mean pH exhibited a minor, albeit insignificant, increase during testing with chloramines (8.15 ± 0.09) as compared to without disinfectant (7.96 ± 0.28). The mean conductivity during testing with chloramines (143.7 ± 1.8 μS/cm) also was slightly elevated compared to that without disinfectant (133.2 ± 3.4 μS/cm). The mean turbidity of the water following periods of stagnation (0.61 ± 0.64 NTU) was greater and more variable than that of flowing water after 8 min of shower operation (0.30 ± 0.11 NTU). Finally, the mean TOC concentration of all samples was 2.8 ± 0.4 mg/L.

When chloramines were dosed, the total chlorine concentration in the mixing tank (Figure 1) was 0.9 ± 0.1 mg/L Cl_2_ (target = 1.0 mg/L Cl_2_), and the average concentrations in the cold and hot water pipes when the water was flowing were 0.8 ± 0.1 mg/L Cl_2_ and 0.4 ± 0.1 mg/L Cl_2_, respectively. However, total chlorine concentrations in stagnant water were consistently at or below the lower limit of detection (0.1 mg/L Cl_2_). Chlorine decay within the pilot system pipes (hot and cold) was compared with that in bulk water incubated in clean glass bottles to assess the role of premise plumbing in chlorine consumption (Figure S4). The first-order decay rates (*K*, summarized in Table S7) inside the hot and cold water pipes (3.76 and 1.16 h^–1^, respectively) were substantially higher than the respective decay rates in the glass bottles (0.03 and 0.01 h^–1^, respectively), suggesting that biofilms or other constituents in the pipes accelerated chloramine decay.

### 3.2 Concentrations of Total Bacteria, *Legionella* spp., and Amoebas in the System

In this work, total bacteria (as 16S rRNA genes), *Legionella* spp. (as *ssrA*), *L. pneumophila* (as *mip*), and *V. vermiformis* (as 18S rRNA genes) were quantified by qPCR (Figure 2). Flow cytometric measurements of total and intact cell counts (Figure S5) correlated strongly with the total bacterial 16S rRNA gene copy numbers (Figure S6; Pearson correlations >0.9), so we primarily focus on the qPCR results. During experiments without chloramines, concentrations of total bacteria during stagnation were similar in the hot and cold water supplies, ranging from 7.9 to 9.4 log_10_[copies/L] (median 8.8 log_10_[copies/L] for both hot and cold water). Comparable results were obtained during experiments with chloraminated water (7.0 to 9.6 log_10_[copies/L]; median 8.9 log_10_[copies/L] for both hot and cold water). Model-based estimates of immediate effects are summarized as marginal means (Table S8) and average marginal effects (Table S9). Flushing decreased total bacteria concentrations by about 1 log_10_[copies/L] for cold water and 2 log_10_[copies/L] for hot water, with similar results in the presence and absence of chloramines. Total bacteria concentrations largely recovered within 2–3 days of postflush stagnation.

**Figure 2:**
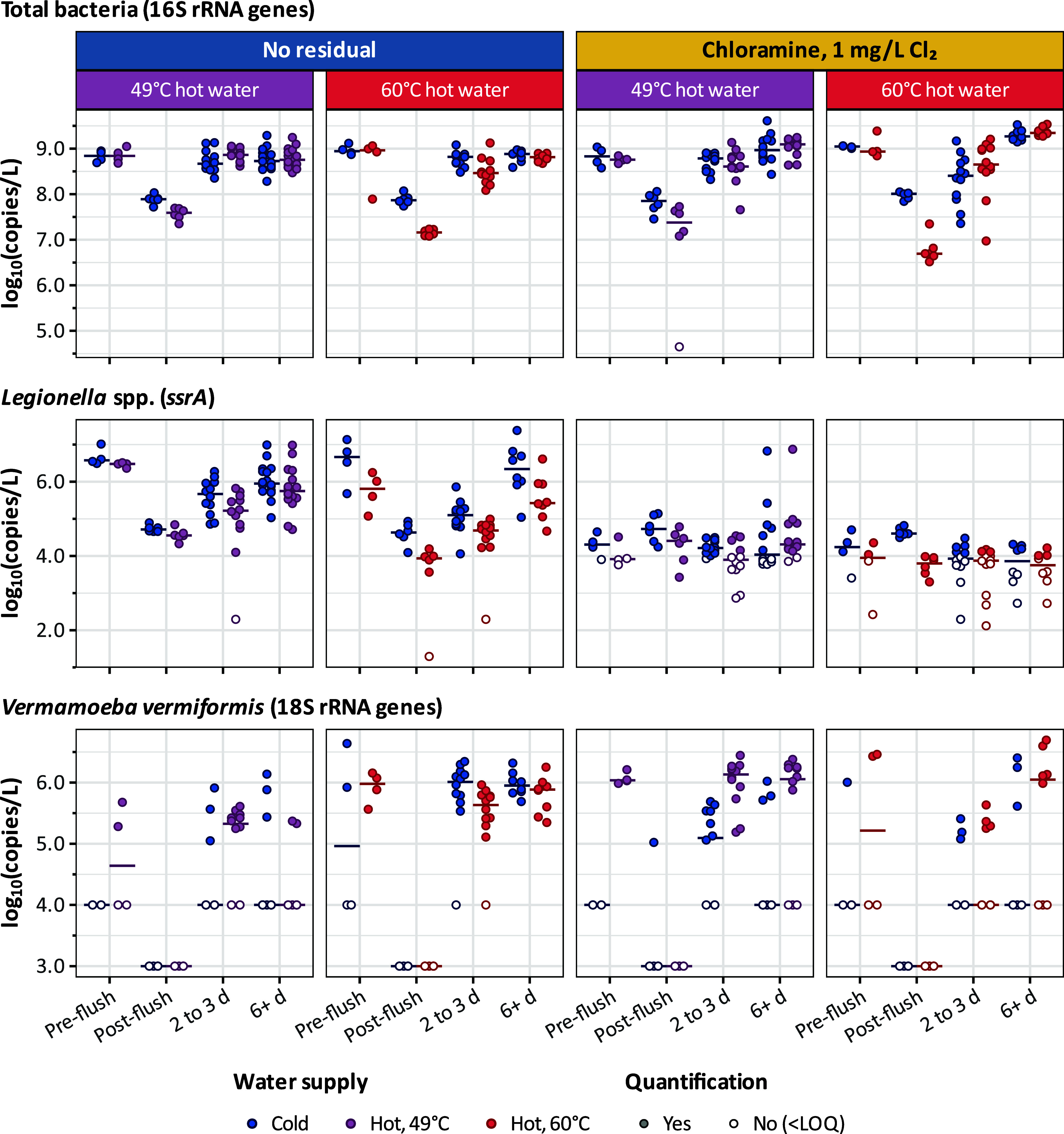
Monitoring of total bacteria, *Legionella* spp., and *V. vermiformis* concentrations in water via qPCR during pilot flushing experiments: pre- and postflush and then after stagnation at 2–3 days and again at 6 or more days. Cold water was collected alongside hot water produced at either 49 °C (1:1 ratio) or 60 °C (7:3 hot/cold ratio) via thermostatic mixing valve. Observations below the LOQ plotted at the sample LOQ, which varied by sample volume.

For *Legionella* spp., concentrations in the absence of chloramines were similar in the hot and cold water supplies during stagnation, ranging from <2.3 to 7.4 log_10_[copies/L] (medians 5.5 log_10_[copies/L] for hot water and 5.9 log_10_[copies/L] for cold). Flushing then decreased the concentrations by about 2 log_10_[copies/L] for both cold and hot water, which recovered at a slower rate than total bacteria (i.e., after about 1 week of postflush stagnation). Unlike total bacteria, the concentrations of *Legionella* spp. during stagnation were substantially lower during experiments with chloraminated water, ranging from <2.1 to 6.9 log_10_[copies/L] for both cold and hot water supplies (medians 3.9 log_10_[copies/L] for hot water and 4.2 log_10_[copies/L] for cold). Furthermore, flushing with chloraminated water resulted in little or no immediate change in *Legionella* spp. concentrations because preflush levels were already low. Nevertheless, during subsequent postflush or postshower stagnation, *Legionella* spp. concentrations increased only slowly, with detectable rebound occurring after approximately 3 weeks—substantially later than the recovery observed for total bacteria. *L. pneumophila*was never detected.

*V. vermiformis* 18S rRNA genes were frequently detected in stagnant water with or without chloramine but rarely in fresh tap water after a shower or flush (Table S10), suggesting amoebas were more prevalent when biomass was elevated or after interaction with pipe biofilms, e.g., via detachment. In cold water, 18S rRNA genes were quantifiable in 18/48 stagnant samples but only 1/24 postshower or flush samples, with concentrations up to 6.6 log_10_[copies/L]. Interestingly, there was more frequent detection of *V. vermiformis* in the stagnant hot water (32/48) than cold water, with concentrations up to 6.7 log_10_[copies/L], despite never being detected in hot water collected during a shower or flush (0/24). Flushing decreased *V. vermiformis* levels to below detection in most cases, but the concentrations recovered within 2–3 days of postflush stagnation. Assessing the effects of disinfectant or pipe material on amoeba levels was precluded by the large fraction of samples that were below the quantification limit.

### 3.3 Comparison of Flushing vs Showering

Model-based analyses of within-event log_10_ concentration changes indicated no meaningful difference between flushing and showering for either total bacteria or *Legionella* spp. in water (Tables S8 and S9). Both interventions reduced total bacterial concentrations by approximately 1–2 log_10_ (ca. 90–99%), while *Legionella* spp. were typically reduced by 0.6–1.0 log_10_ (ca. 70–90%), depending on experimental conditions.

Across all models, the presence of chloramines was associated with greater immediate reductions in total bacteria but smaller apparent reductions in *Legionella* spp. This pattern primarily reflected substantially lower pre-event *Legionella* concentrations in chloraminated water rather than reduced removal efficiency during flushing or showering. Consistent with this interpretation, marginal effect estimates showed no evidence that flushing outperformed showering as a flow-based intervention for reducing planktonic bacterial concentrations in water.

### 3.4 Effect of Hot Water Temperature on Flushing Effectiveness

Postflush concentrations of total bacteria and *Legionella* spp. were generally higher in cold water and 49 °C hot water compared to 60 °C hot water. Model-based estimates indicated a modest but statistically supported effect of higher hot-water temperature on immediate reductions of total bacteria, whereas effects on *Legionella* spp. were weak and not consistently significant (Tables S8 and S9).

### 3.5 Effect of Pipe Material on Bacteria Levels in the Water and Biofilms

Model-based analyses provided insufficient evidence that pipe material (copper versus Aqua PE-Xa) influenced immediate reductions or stagnant-water concentrations of total bacteria or *Legionella* spp. in water when accounting for disinfectant presence and temperature (Tables S8 and S9). Similarly, biofilm-associated concentrations of total bacteria and *Legionella* spp. did not differ between copper and PE-Xa pipes.

### 3.6 Chloramines Delay Increases in *Legionella* spp. but not Total Bacteria during Postflush/Shower Stagnation

Total bacteria levels increased at a relatively rapid rate during stagnation (median μ_max_ of 1.3/day; Table S11) and typically plateaued within 1–4 days (*t*_max_), regardless of the starting concentration after a flush or shower (Figure 3). Chloraminated water, for example, had lower starting total bacterial biomass than water with no residual disinfectant, and cold water had greater starting biomass than hot water. Nevertheless, the ending bacterial 16S rRNA gene concentrations were similar for all cold and hot water samples, although slightly higher for chloraminated water (9.0 to 9.3 log_10_[copies/L]) than for water without residual disinfectant (8.8 to 8.9 log_10_[copies/L]).

**Figure 3:**
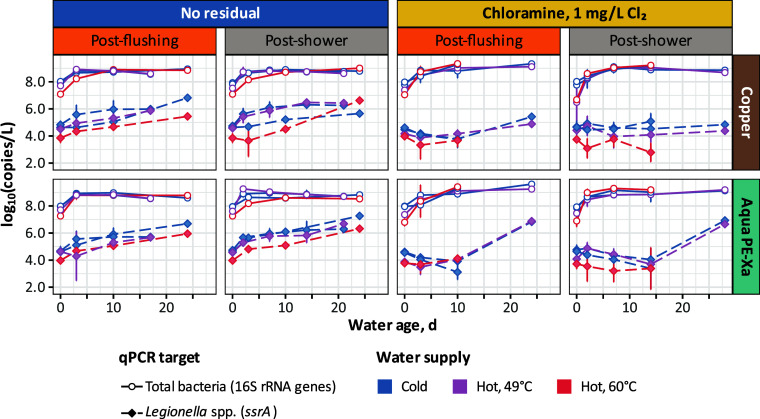
Total bacteria (16S rRNA gene copies) and *Legionella* spp. (*ssrA* copies) in water vs water age in stagnant copper or Aqua PE-Xa pipes after either flushing at maximum flow rate for 5 min or replacing the water with a simulated shower.

Unlike total bacteria, *Legionella* spp. were significantly impacted by the presence of chloramines and hot water temperature set-point (Figure 3). Apparent growth of *Legionella* spp., as inferred from increases in *ssrA* gene copy numbers, was significantly delayed compared to no residual disinfectant, with little or no increase observed over the first 10–14 days of stagnation. Elevated levels of *Legionella* spp. were sometimes observed after 24 or more days following a flush or shower with chloraminated water, and interestingly, only in the PE-Xa pipes.

### 3.7 Effect of Flushing on Biofilms

Compared to biofilms in the stagnant pipes, the concentrations of total bacteria (as 16S rRNA genes; Figure S7) were significantly lower in biofilms after flushing (adjusted *p* = 0.003 using the Wilcoxon signed rank test) and after showering (adj. *p* = 0.004). However, total bacteria concentrations in biofilms after flushing (maximized flow rate) were not significantly different from those after showering (conventional flow rate) (adj. *p* = 0.081). Conversely, compared to biofilms in the stagnant pipes, the concentrations of *Legionella* spp. (as *ssrA*; Figure S8), decreased significantly only after flushing (adj. *p* = 0.003), but not after showering (adj. *p* = 0.87). Thus, flushing enhanced the removal of *Legionella* in pipe biofilms compared to showering (adj. *p* = 0.046).

While there was occasionally low-level amplification of the 18S rRNA gene target for *V. vermiformis*, all observations of biofilm samples fell below the method quantification limit. Additionally, *L. pneumophila* mip genes were never detected in biofilm samples.

## 4 Discussion

Pilot-testing was conducted to compare the effects of flushing, periodic showering, and extended stagnation on total bacteria and *Legionella* spp. concentrations in premise plumbing. Using a controlled pilot-scale system, this study revealed novel insights into how hot-water temperature, chloramine disinfectant, and two types of pipe material influence the effectiveness of flushing or showering in controlling total bacteria and *Legionella* spp. in both water and biofilms.

Flushing and showering were comparable in terms of their effects on total bacterial biomass in water and biofilm and on *Legionella* spp. in the water. The rebound of total bacteria concentrations in the water during postflush stagnation and postshower stagnation were similar, as well as for *Legionella*. Flushing resulted in higher velocities through the pipes and hence greater shear stress, which might be expected to increase biofilm removal.^55,56^ Increased biofilm removal might, in turn, slow bacterial rebound in bulk water by limiting the input of detached biofilm cells. Flushing resulted in a minor yet statistically significant decrease of *Legionella* spp. from pipe wall biofilms compared to showering but did not slow the rebound in the bulk water. Overall, our findings suggest that flushing provided limited additional benefit in comparison to conventional shower operation (i.e., leaving the showerhead and any tubing intact and initiating water flow). Thus, it appears that simply replacing the older water with “fresh” water from the mains provides a similar benefit regardless of flow velocity and that the additional effort of removing flow-restricting devices such as showerheads may not be worthwhile. However, removal of the showerhead, and tubing if applicable, does afford the opportunity to clean them which may provide additional benefits in terms of limiting *Legionella* spp. exposure.

From a practical perspective, these findings suggest that under routine occupancy conditions—where fixtures are used daily and stagnant water is regularly displaced—normal shower operation may provide benefits comparable to flushing. In contrast, flushing may remain justified following extended stagnation,^6–9^ such as during building recommissioning, low-occupancy periods, or prior to reoccupancy, when water turnover has been minimal and microbial accumulation is more likely. In such scenarios, flushing can serve as an initial intervention to replace aged water, although the present results indicate that sustained *Legionella* management and risk mitigation depends more strongly on restoring regular water use than on high-velocity flushing alone. These observations help contextualize prior flushing recommendations by emphasizing the importance of water turnover frequency rather than flushing intensity.

There is strong evidence supporting the effectiveness of chloramines against *Legionella* in building water systems, even without high frequency flushing or water use to consistently maintain a residual.^57,58^ Our results suggest that daily exposure to chloramines via flushing or showering may be sufficient to keep *Legionella* under control. The necessary exposure frequency likely will depend on the type of disinfectant. Totaro et al.^59^ observed a decrease in *L. pneumophila* in contaminated hospital water systems using free chlorine, but only when using electronic taps that self-flushed for 1 min every 2 h (approximately 192 L/day per tap). This difference in the necessary exposure frequency may be due to the higher reactivity of free chlorine compared to monochloramine, which results in a shorter half-life and limited penetration into biofilms.^20,60–63^

Although a switch from free chlorine to chloramines in municipal water supplies has been associated with decreases in *Legionella* levels in buildings,^57^ it remains uncertain whether this effect persists over the long-term. Free chlorine may suppress *Legionella* more effectively in the distribution network,^64^ but less effective in buildings, which complicates direct comparisons between disinfectants at different points and water ages in the network. While Flannery et al.^58^ observed that the decrease attributed to chloramines persisted for at least 2 years, other local factors might counteract the benefits of chloramines. For instance, Wang et al.^65^ found that simulated hot water heaters receiving chloraminated water had more *Legionella* compared to those receiving chlorinated water due to rapid chloramine decay via nitrification. Moreover, bacterial communities can develop increased resistance to disinfectants over time.^66^ Hozalski et al.^6^ reported that *Legionella* spp. were observed in 20% of flushed showers within 2–4 days and 40% within 6–7 days. Although the rebound occurred much faster in their study than observed here (i.e., >10 days for experiments with chloraminated water), the full-scale distribution system sampled by Hozalski et al.^6^ had employed chloramines as residual disinfectant for several decades. Thus, that full-scale system may have contained a *Legionella* population better adapted to chloramines than the pilot system in the present study. These studies collectively indicate that while chloramines may initially lead to reductions in *Legionella*, various factors, including chloramine decay and bacterial adaptation, might influence its effectiveness in controlling *Legionella*.

In the present study, the response of total bacterial biomass to flushing or showering and its recovery during postflush or postshower stagnation was largely unaffected by the presence of chloramine. Certain subpopulations of a diverse drinking water microbiome tend to be better adapted to handle specific environmental stressors. Thus, complex bacterial communities can adapt to stressors like chemical disinfectants, often by shifting community composition in favor of resistant populations. This phenomenon is evident in chloraminated drinking water distribution networks, where biofilms can shift toward chloramine-resistant mycobacteria and ammonia-oxidizing bacteria.^67–70^ A shift in suspended bacterial communities was also observed by Baron et al.^71^ after onsite chloramination in a hospital hot water system.

A water temperature of 50 °C is often recognized as the upper tolerance for *Legionella* growth and culturability.^72^ Beyond this threshold, *Legionella* may persist in a viable but nonculturable (VBNC) state—potentially up to pasteurization-capable temperatures (70 °C or above).^73–75^ In the United States, water heaters are commonly set at 120 °F (49 °C), per guidance from the U.S. Environmental Protection Agency and U.S. Department of Energy, to decrease the risk of scalding in addition to energy savings. However, with thermostats set to less than 50 °C, there is greater risk of *Legionella* proliferation at distal taps under low-frequency water use conditions.^76^ Recognizing this risk, the same U.S. agencies recommend setting water heater thermostats at 140 °F (60 °C) in healthcare settings or in homes with high-risk individuals. The Norwegian Institute of Public Health recommends that water heater thermostats be set at a minimum of 70 °C,^27^ and water heaters sold in Norway typically are preset by the manufacturer at 75 °C. In addition, hot water should be at least 60 °C throughout the hot water lines with thermostatic mixing valves or other fixture features used to reduce water temperature at the point of use to prevent scalding.^27^ Hence, to achieve a target of 60 °C in hot water lines and 40 °C at the point of use requires blending with cold water for hot water heaters set at temperatures sufficient to inactivate legionellae.

Flushing with water at 60 °C as a form of heat-shock treatment may provide immediate reductions in *L. pneumophila*, but such effects are often short-lived.^77^ Epalle et al.^78^ further demonstrated that *L. pneumophila* rendered VBNC by 30 min of heat treatment at 70 °C could still be resuscitated in host amoebas. In the present study, the internal water-heater temperature was set to 85 °C to ensure sufficient hot-water capacity during repeated flushing and showering, whereas the temperatures delivered to the rig piping were controlled by thermostatic mixing at 49 or 60 °C. Thermal inactivation is strongly time dependent,^75,79^ and exposure conditions differed between biofilm-associated and planktonic cells in the pilot. Biofilms lining the distal piping were exposed to 49 or 60 °C for the full duration of flushing (5 min) or showering (approximately 8 min). In contrast, planktonic cells originating from the cold-water supply were mixed with hot water at the thermostatic mixing valve and experienced peak temperatures of 49 or 60 °C only during transport to the outlets, after which the mixed water cooled toward ambient temperature during stagnation. Given reported *Legionella* inactivation kinetics at 60 °C,^79,80^ such exposure is likely insufficient to reliably inactivate planktonic cells introduced from the cold-water supply. Consequently, cold water likely represents the primary source of *Legionella* entering hot and distal water lines following blending. Conversely, when chloramines are present, the cold-water supply is also expected to contribute most of the residual disinfectant to blended hot water, as chlorine-based disinfectants decay rapidly at elevated temperatures in water heaters.^6,81^

When examining the impact of elevated temperature in the absence of chloramine, we observed some regrowth of *Legionella* following a flush or shower with the hot water set at 60 °C. However, the higher hot water temperature seemed to prolong the lag phase and slow down the rate of growth. Previous studies have shown that implementing a good thermal regime, such as weekly flushing of hot water lines, maintaining the hot water at 60 °C, and ensuring at least 55 °C throughout the hot-water lines, can decrease the abundance of *L. pneumophila*.^82^ However, *L. pneumophila* has been reported to be more resistant to thermal inactivation compared to non-*pneumophila Legionella* spp.^83^ Studies have also indicated that higher temperatures, such as 70 °C, could select for thermal resistance in *L. pneumophila* over time, and some *Legionella* species, like *L. thermalis*, are well-adapted to hot spring water and other hot environments.^74,84^ Thus, careful management of hot water temperatures throughout building plumbing systems may be insufficient for controlling *Legionella* spp. in the absence of residual disinfectant.

In general, the type of distal piping (i.e., copper versus PE-Xa) did not have a significant impact on the concentrations of total bacteria in the bulk water or biofilms. The only exception was the enhanced regrowth of *Legionella* spp. in the PE-Xa pipes following flushing or showering with chloraminated water, but only after more than 3 weeks of stagnation. The enhanced regrowth in the PE-Xa pipes at long stagnation times might be due to the tendency of plastic materials to release soluble organic compounds into the water that could serve as substrates for bacterial growth.^85^ Conversely, copper pipes can release cupric ion (Cu^2+^) which has well-known biocidal capabilities.^86,87^ However, the biocidal efficacy of (Cu^2+^) depends on environmental conditions, including pH and NOM concentration, and the *L. pneumophila* growth phase.^88^ As observed in the present study, the release of Cu^2+^ decreases over time as protective scale layers develop, especially with use of corrosion inhibitors.^88^ Buse et al.^20^ observed that disinfectant type and pipe material were important interrelated factors for the survival of *L. pneumophila*. Nonetheless, our limited results suggest that pipe material was not a major factor, except perhaps over long periods of stagnation.

A limitation of this study is that *Legionella* dynamics were assessed using qPCR, which quantifies gene copy numbers and does not distinguish between viable, nonviable, or extracellular DNA. Consequently, increases in *ssrA* gene copy numbers cannot be interpreted as definitive evidence of cellular replication. Nonetheless, the observed lag phases, growth-rate differences, and treatment-dependent temporal patterns are consistent with biological growth responses reported in prior studies and support the interpretation that chloramines and elevated temperature suppress *Legionella* proliferation under the conditions tested.

In summary, environmental stressors like chloramine or elevated temperature generally aid in controlling *Legionella*, and our findings align with previous research, suggesting that using flushing to maintain these stressors can effectively manage *Legionella*. Importantly, the results also underscore the central role of stagnation in promoting microbial rebound, indicating that regular water turnover—rather than high flow velocities or aggressive flushing protocols—may be the primary factor in limiting microbial regrowth in premise plumbing systems. The combination of these mitigation efforts may compound the beneficial effects. It is important to note, however, that bacteria are living organisms subject to selective forces and capable of adaptation. Therefore, further research is necessary to understand whether long-term exposure to stressors could lead to the selection of more resistant *Legionella* strains and contribute to unforeseen, persistent problems.

## Supplementary Material



## Notes

Linear model specification and selection; municipal tap water and pilot feedwater quality; primary experimental variables and hydraulic characteristics during flushing and showering; qPCR primers, probes, and calibration data (including oligonucleotide standard sequences); experimental design and sampling cycles; flow cytometry gating strategy and comparison of flow cytometric cell counts with qPCR-derived total bacteria; chloramine decay kinetics; model-based summaries of immediate qPCR responses to outlet operation (marginal mean log reductions and average marginal effects) and detection frequency of *Vermamoeba vermiformis*; estimated bacterial growth model parameters during stagnation; qPCR results for total bacteria; and *Legionella* in pipe biofilms (PDF)
