# Supplementary material for: Flushing as a Control Measure for Legionella spp.: Impacts of Water Age, Chloramine Disinfection, and Elevated Temperature
Source: Environ Sci Technol. 2026 Jan 28;60(5):3935–46. doi: 10.1021/acs.est.5c16399 (PMC12895531; doi:10.1021/acs.est.5c16399)
Supplement: Supplementary file 1 [file es5c16399_si_001.pdf]

Supporting Information: Flushing as a Control Measure for *Legionella* spp.: Impacts of Water Age, Chloramine Disinfection, and Elevated Temperature

Charuka S. Meegoda,† Michael B. Waak,*,†,‡ Taegyu Kim,¶
Raymond M. Hozalski,¶ and Cynthia Hallé†

†Department of Civil and Environmental Engineering, Norwegian University of Science and Technology, 7031 Trondheim, Norway

‡Department of Infrastructure, SINTEF Community, 7031 Trondheim, Norway

¶Department of Civil, Environmental, and Geo-Engineering, University of Minnesota, Minneapolis, Minnesota, 55455, United States

E-mail: michael.waak@sintef.no

19 pages: supporting text, 11 supporting tables (S1 to S11), and 8 supporting figures (S1 to S8)

Supporting Text

Linear model specification and selection

Linear models with additive main effects (Eq. 1) were compared against models including all two- and three-way interactions among factors (Eq. 2) using Akaike's information criterion (AIC).

Across all water-phase analyses, additive models were consistently favored over interaction models and were therefore retained for inference, with strong AIC weight support[S1] (Model A, $w_{\mathrm{AIC}} = 0.74$; Model B, $w_{\mathrm{AIC}} = 1.00$; Model C, $w_{\mathrm{AIC}} = 0.92$; Model D, $w_{\mathrm{AIC}} = 0.98$). For hot-water analyses (Models B and D), hot-water temperature set-point (49 °C or 60 °C) was included as an additional additive main effect; interaction terms were not supported.

$$Y = \beta_0 + \beta_1 X_1 + \beta_2 X_2 + \beta_3 X_3 + \epsilon \quad (1)$$

$$Y = \beta_0 + \beta_1 X_1 + \beta_2 X_2 + \beta_3 X_3 + \beta_4 X_1 X_2 + \beta_5 X_1 X_3 + \beta_6 X_2 X_3 + \beta_7 X_1 X_2 X_3 + \epsilon \quad (2)$$

Where:

- Y is the dependent variable (response), defined as the within-event change in $\log_{10}$-transformed qPCR gene target concentration (post- minus pre-water use), i.e., the immediate $\log_{10}$ reduction
- β_0 is the model intercept, representing the expected $\log_{10}$ reduction at the reference levels of all categorical predictors
- X_1 is the categorical predictor representing outlet operation (flushing versus showering)
- X_2 is the categorical predictor representing disinfectant condition (chloramine present versus absent)
- X_3 is the categorical predictor representing pipe material (copper versus Aqua PE-Xa)
- $\beta_1, \beta_2, \beta_3$ are regression coefficients representing the main effects of outlet operation, disinfectant, and pipe material, respectively
- $\beta_4, \beta_5, \beta_6$ are coefficients for the two-way interaction terms between predictors
- β_7 is the coefficient for the three-way interaction among outlet operation, disinfectant, and pipe material
- ϵ is the residual error term, assumed to be independently and normally distributed with mean zero and constant variance

Table S1: Water quality of municipal tap water and pilot feed water. Adapted with permission from Waak et al.[S2] Copyright 2024 The Water Research Foundation.

Parameter	Unit	Median	Range	Reference
**Municipal tap water**				
Temperature	°C	4.1	1.0 – 5.5	City of Trondheim[S3]
pH		8.1	7.8 – 8.6	City of Trondheim[S3]
Hardness*	mg/L $CaCO_3$	54	53 – 55	City of Trondheim[S3]
Alkalinity	mg/L $CaCO_3$	105		City of Trondheim[S3]
Conductivity	$\mu S/cm^2$	128	113 – 137	City of Trondheim[S3]
Turbidity	FNU	0.1	0.1 – 3.6	City of Trondheim[S3]
TOC	mg/L C	3.0	2.0 – 4.0	City of Trondheim[S3]
AOC (P-17/NOX)	µg/L C	118	72 – 268	Waak et al.;[S4] Johansen[S5]
Ammonium, NH_4^+	mg/L N	0.005		City of Trondheim[S3]
Nitrate, NO_3^-	mg/L N	1.9	0.9 – 2.5	City of Trondheim[S3]
Nitrite, NO_2^-	mg/L N	0.003		City of Trondheim[S3]
Phosphorus, total	mg/L P	< 0.001		Waak et al.[S4]
Chlorine, total	mg/L Cl	0.07	0.05 – 0.12	City of Trondheim[S3]
Copper	µg/L Cu	45	3 – 87	City of Trondheim[S3]
Sulfate, SO_4^{2-}	mg/L S	0.91	0.89 – 0.92	City of Trondheim[S3]
Iron, total	µg/L Fe	5.1	2.0 – 6.9	City of Trondheim[S3]
**Pilot feed water via laboratory plumbing**				
Temperature	°C	10.0	7.6 – 13.6	This work
pH		8.0	7.7 – 8.3	This work
Conductivity	$\mu S/cm^2$	129	124 – 140	This work
Turbidity	FNU	0.6	0.2 – 4.4	This work
TOC	mg/L C	2.7	2.5 – 3.0	This work
AOC (P-17/NOX)	µg/L C	96	28 – 244	Waak et al.;[S4] Johansen[S5]
Chlorine, total	mg/L Cl	< 0.02		This work
Chlorine, total (NH_2Cl added)	mg/L Cl	< 0.02		This work
Copper	µg/L Cu	180	48 – 1270	This work

* Water hardness estimated from Ca^{2+} and Mg^{2+} concentrations.

Table S2: Primary experimental variables and their rationale

Variable	Values	Rationale
Disinfectant residual	None	Absent or depleted residual
	Chloramines, 1 mg/L Cl_2	Microorganism suppression
Hot-water temperature	49 °C (120 °F)	Scald prevention, energy conservation
	60 °C (140 °F)	*Legionella* prevention
Pipe material	Copper	Commonly-used metal
	Uponor Aqua PE-Xa	Commonly-used plastic
Control measure (flow rate)	None	Conventional water use
	Flushing (max. flow, 5 min)	*Legionella* prevention

Table S3: Summary of hydraulic characteristics in the distal cold- and hot-water branches during flushing and showering. Adapted with permission from Waak et al.[S2] Copyright 2024 The Water Research Foundation.

Operation	Supply	Material	Temperature, °C			Flow rate, L/min			Velocity, m/s			Reynolds number			Shear stress, N/m^2		
			median	min	max	median	min	max	median	min	max	median	min	max	median	min	max
**Hot-water setpoint at 49 °C**																	
Flush	Cold	Copper	9.0	6.7	12.8	14.9	10.3	19.1	2.02	1.39	2.59	20 900	12 800	23 000	13.25	7.07	21.28
		PE-Xa	8.9	8.1	13.3	15.6	10.6	17.1	1.29	0.88	1.41	16 400	10 400	17 300	5.64	2.97	6.84
	Hot	Copper	46.9	46.3	47.6	11.3	9.8	13.8	1.53	1.34	1.88	33 100	28 900	40 000	6.78	5.31	9.76
		PE-Xa	48.1	47.3	48.6	12.4	11.4	13.8	1.03	0.94	1.14	28 700	26 600	32 400	3.15	2.68	3.78
Shower*	Blend	Copper	40.1	37.8	42.1	7.4	6.7	10.9									
		PE-Xa	39.0	37.0	40.7	7.5	6.5	12.0									
	Cold	Copper	12.8	7.9	12.8	1.4	1.0	1.9	0.19	0.14	0.26	1980	1280	2690	0.15	0.13	0.24
		PE-Xa	13.2	8.6	13.2	2.0	1.7	2.5	0.17	0.14	0.21	2140	1620	2790	0.11	0.08	0.16
	Hot	Copper	47.3	46.5	47.3	5.9	5.4	9.7	0.80	0.73	1.32	17 100	15 800	28 200	2.13	1.82	5.23
		PE-Xa	48.2	47.9	48.2	5.5	4.8	9.8	0.46	0.40	0.81	12 800	11 200	22 600	0.74	0.60	2.05
**Hot-water setpoint at 60 °C**																	
Flush	Cold	Copper	9.7	6.1	12.5	16.7	16.5	18.7	2.27	2.24	2.53	21 600	21 300	23 100	16.42	15.98	20.59
		PE-Xa	7.6	6.4	8.4	15.7	15.0	18.9	1.30	1.24	1.56	15 200	14 200	17 200	5.89	5.45	8.26
	Hot	Copper	59.6	57.2	60.6	13.6	13.2	13.8	1.84	1.79	1.87	48 600	45 300	49 300	8.94	8.56	9.17
		PE-Xa	59.8	57.2	60.6	14.0	12.9	16.3	1.16	1.07	1.35	39 200	34 700	45 600	3.72	3.24	4.87
Shower*	Blend	Copper	41.0	38.8	42.4	7.2	6.7	13.5									
		PE-Xa	41.0	39.0	43.1	7.3	6.4	12.9									
	Cold	Copper	10.6	6.1	10.6	2.7	2.3	5.0	0.37	0.32	0.68	3560	3090	6580	0.60	0.39	1.99
		PE-Xa	8.0	6.4	8.0	2.6	2.0	4.4	0.22	0.16	0.36	2500	1870	4200	0.16	0.11	0.65
	Hot	Copper	60.0	57.2	60.0	4.8	4.0	8.6	0.65	0.55	1.16	17 000	14 500	30 700	1.39	1.05	3.94
		PE-Xa	60.0	57.2	60.0	4.9	4.2	9.0	0.41	0.34	0.75	13 800	11 700	24 300	0.59	0.44	1.72

* Temperature and flow rate were measured for the blended water (ca. 40 °C), and temperature was measured in the cold and hot water (i.e., outlet temperature at full cold or hot). Flow rate for the cold and hot water pipes (prior to blending) was estimated by a thermal mass balance with the blended water, and then the velocity, Reynolds number, and shear stress were subsequently calculated from flow rate.

Table S4: qPCR primers, probes and protocols

**Target**		**Primer/probe sequence (5’ to 3’)**[*]	**Conc., nM**	**Amplicon**	**PCR protocol**
**Domain *Bacteria***	341F	CCT ACG GGA GGC AGC AG	500	ca. 200 bp	1 min at 95 °C; 30 cycles of 95 °C
16S rRNA genes, V3 region[S6]	534R	ATT ACC GCG GCT GCT GG	250		for 15 s and 60 °C for 1 min
**Genus *Legionella***	PanLegF	GGC GAC CTG GCT TC	500	101 bp	10 min at 95 °C; 45 cycles of 95 °C
ssrA[S7]	PanLegR	GGT CAT CGT TTG CAT TTA TAT TTA	500		for 15 s and 60 °C for 1 min
	PanLegP	6-FAM/ACG TGG GTT GCA A/MGBNFQ	100		
Legionella pneumophila	LpF	TTG TCT TAT AGC ATT GGT GCC G	500	115 bp	10 min at 95 °C; 45 cycles of 95 °C
mip[S7]	LpR	CCA ATT GAG CGC CAC TCA TAG	500		for 15 s and 60 °C for 1 min
	LpP	6-FAM/CGG AAG CAA/ZEN/TGG CTA AAG GCA TGC A/IBFQ	100		
Vermamoeba vermiformis	Hv1227F	TTA CGA GGT CAG GAC ACT GT	500	502 bp	3 min at 95 °C; 45 cycles of 95 °C
18S rRNA genes[S8]	Hv1728R	GAC CAT CCG GAG TTC TCG	500		for 20 s and 58 °C for 30 s and 72 °C for 40 s

[*] Abbreviations: 6-FAM, 6-FAM™ fluorescein reporter dye; MGBNFQ, TaqMan® minor groove binder non-fluorescent quencher; ZEN, ZEN™ internal fluorescence quencher; IBFQ, Iowa Black® dark quencher.

Table S5: Template sequences for qPCR oligonucleotide standards

Taxon (PCR target)	GenBank reference genome	Accession no.	Position (length)
Total bacteria (16S rRNA gene)	*Escherichia* sp. UIWRF0630 16S ribosomal RNA gene, partial sequence	KR190116.1	241 to 467 (227 bp)
Legionella spp. (*ssrA*)	*Legionella pneumophila* strain Philadelphia 1 ATCC, complete genome	CP015927.1	172901 to 173032 (132 bp)
L. pneumophila (*mip*)	*Legionella pneumophila* strain Philadelphia 1 ATCC, complete genome	CP015927.1	915183 to 915327 (145 bp)
V. vermiformis (18S rRNA gene)	*Hartmannella vermiformis* 18S ribosomal RNA gene, complete sequence	AF426157.1	1212 to 1743 (532 bp)

Table S6: Summary of real-time qPCR standards and calibration curves

Assay	Standards*			Calibration curve†			
	no.	low	high	% eff.	R^2	slope	intercept
**16S rRNA genes (total bacteria)**							
1	7	1×10^2	1×10^8	99.3	0.997	−3.34	38.5
2	7	1×10^2	1×10^8	99.6	0.994	−3.33	38.0
3	7	1×10^2	1×10^8	99.8	0.997	−3.33	37.6
4	7	1×10^2	1×10^8	99.3	0.996	−3.34	37.6
5	7	1×10^2	1×10^8	99.5	0.994	−3.33	37.8
6	7	1×10^2	1×10^8	100.0	0.997	−3.32	37.7
7	7	1×10^2	1×10^8	96.0	0.996	−3.42	38.0
8	7	1×10^2	1×10^8	98.0	0.998	−3.37	36.8
9	7	1×10^2	1×10^8	94.4	0.998	−3.46	37.5
***ssrA* (*Legionella* spp.)**							
1	7	1×10^1	1×10^7	100.1	0.994	−3.32	41.2
2	7	1×10^1	1×10^7	98.9	0.998	−3.35	40.9
3	7	1×10^1	1×10^7	106.2	0.999	−3.18	39.7
4	7	1×10^1	1×10^7	106.4	0.999	−3.18	40.0
5	7	1×10^1	1×10^7	103.6	0.991	−3.24	40.5
6	7	1×10^1	1×10^7	107.2	0.998	−3.16	39.9
***mip* (*Legionella pneumophila*)**							
1	7	1×10^1	1×10^7	99.0	0.999	−3.35	40.5
2	7	1×10^1	1×10^7	106.4	0.998	−3.18	39.5
3	7	1×10^1	1×10^7	101.6	0.998	−3.28	38.1
4	7	1×10^1	1×10^7	103.4	0.995	−3.24	40.6
5	7	1×10^1	1×10^7	107.5	0.993	−3.16	40.3
6	7	1×10^1	1×10^7	107.2	0.998	−3.16	39.9
7	7	1×10^1	1×10^7	105.2	0.998	−3.20	39.5
**18S rRNA genes (*Vermamoeba vermiformis*)**							
1	7	1×10^1	1×10^7	83.5	0.999	−3.79	37.0
2	7	1×10^1	1×10^7	86.6	0.999	−3.69	40.0
3	7	1×10^1	1×10^7	86.8	0.999	−3.68	39.4
4	7	1×10^1	1×10^7	83.1	0.999	−3.81	37.3
5	7	1×10^1	1×10^7	87.0	0.999	−3.68	37.1
6	6	1×10^1	1×10^7	96.3	1.000	−3.41	35.1
7	7	1×10^1	1×10^7	91.2	1.000	−3.55	35.9
8	7	1×10^1	1×10^7	103.9	0.998	−3.23	33.5
9	7	1×10^1	1×10^7	84.6	0.997	−3.76	36.2

* Number of standards and concentrations of the lowest and highest standards (copies/µL)

† Linear regression of C_q values versus $\log_{10}$-transformed concentrations. Amplification efficiency was calculated as $(10^{-1/\text{slope}} - 1) \times 100\,\%$

Table S7: Chloramine decay constants (*K*) from different water supplies in pipes and glass bottles

**Water supply**	**Milieu**	**Estimate**	**Std. Error**
MilliQ	Bottle	3.0×10^{-3}	8.0×10^{-5}
Cold	Pipe	1.2	1.2×10^{-1}
	Bottle	1.1×10^{-2}	1.2×10^{-3}
Hot	Pipe	3.8	7.7×10^{-1}
	Bottle	3.1×10^{-2}	8.1×10^{-3}

Table S8: Marginal mean* immediate reductions in qPCR gene target concentrations in water before and after outlet operation. Adapted with permission from Waak et al.[S2] Copyright 2024 The Water Research Foundation.

		**Log_{10} reduction†,‡**			**Percent change‡,§**		
**Factor**	**Level**	Mean	LCL	UCL	Mean	LCL	HCL
**Model A: 16S rRNA gene copy number in cold water**							
Intervention	Shower	1.10	1.03	1.18	92.1	90.6	93.4
	Flushing	1.13	1.06	1.21	92.7	91.3	93.8
Disinfectant	None	1.01	0.93	1.08	90.2	88.4	91.8
	Chloramine	1.23	1.15	1.30	94.1	93.0	95.0
Pipe material	Copper	1.11	1.04	1.19	92.3	90.9	93.5
	Aqua PE-Xa	1.12	1.05	1.20	92.5	91.1	93.7
**Model B: 16S rRNA gene copy number in hot water**							
Intervention	Shower	1.81	1.56	2.06	98.5	97.3	99.1
	Flushing	1.90	1.65	2.15	98.7	97.8	99.3
Disinfectant	None	1.46	1.21	1.70	96.5	93.8	98.0
	Chloramine	2.26	2.01	2.51	99.4	99.0	99.7
Pipe material	Copper	2.02	1.77	2.26	99.0	98.3	99.5
	Aqua PE-Xa	1.70	1.45	1.95	98.0	96.5	98.9
Temp. set-point	49 °C	1.67	1.42	1.92	97.8	96.2	98.8
	60 °C	2.05	1.80	2.29	99.1	98.4	99.5
**Model C: *ssrA* copy number in cold water**							
Intervention	Shower	0.71	0.45	0.97	80.4	64.1	89.3
	Flushing	0.56	0.30	0.83	72.6	49.7	85.1
Disinfectant	None	1.71	1.45	1.98	98.1	96.4	98.9
	Chloramine	−0.44	−0.71	−0.18	−176.7	−407.9	−50.8
Pipe material	Copper	0.66	0.40	0.92	78.1	59.9	88.1
	Aqua PE-Xa	0.61	0.35	0.87	75.5	55.1	86.7
**Model D: *ssrA* copy number in hot water**							
Intervention	Shower	0.94	0.57	1.31	88.5	73.3	95.1
	Flushing	1.00	0.63	1.36	89.9	76.6	95.7
Disinfectant	None	1.81	1.44	2.18	98.5	96.4	99.3
	Chloramine	0.13	−0.24	0.49	25.1	−74.0	67.7
Pipe material	Copper	0.73	0.37	1.10	81.5	56.9	92.0
	Aqua PE-Xa	1.20	0.84	1.57	93.8	85.5	97.3
Temp. set-point	49 °C	0.90	0.54	1.27	87.4	70.9	94.6
	60 °C	1.04	0.67	1.40	90.8	78.6	96.0

* Marginal means are model-adjusted estimates averaged over a balanced reference grid for the remaining covariates.
† Negative log reductions indicate higher concentrations in post-use (feed) water than in pre-use (aged) water, reflecting increases following outlet operation.
‡ LCL and UCL = lower and upper 95 % confidence limits for the marginal mean, respectively
§ Percent change is a transformation given by $P = \left(1 - 10^{-R}\right) \times 100\,\%$, where R is the $\log_{10}$ reduction

Table S9: Average marginal effects* of experimental variables (model factors) on immediate $\log_{10}$ reductions of qPCR gene target concentrations in water. Adapted with permission from Waak et al.[S2] Copyright 2024 The Water Research Foundation.

Factor	Contrast	Effect†	SE	Test stat.	*p* value‡	Adj. *p* value‡
**Model A: 16S rRNA gene copy number in cold water**						
Disinfectant	Chloramine − None	0.22	0.05	4.1	<0.01	<0.01
Intervention	Flushing − Shower	0.03	0.05	0.6	0.57	1.00
Pipe material	Aqua PE-Xa − Copper	0.01	0.05	0.1	0.88	1.00
**Model B: 16S rRNA gene copy number in hot water**						
Disinfectant	Chloramine − None	0.80	0.18	4.5	<0.01	<0.01
Intervention	Flushing − Shower	0.09	0.18	0.5	0.62	0.62
Pipe material	Aqua PE-Xa − Copper	−0.32	0.18	−1.8	0.08	0.15
Temp. set-point	60 °C − 49 °C	0.38	0.18	2.1	0.03	0.10
**Model C: *ssrA* copy number in cold water**						
Disinfectant	Chloramine − None	−2.16	0.19	−11.3	<0.01	<0.01
Intervention	Flushing − Shower	−0.15	0.19	−0.8	0.44	0.88
Pipe material	Aqua PE-Xa − Copper	−0.05	0.19	−0.3	0.80	0.88
**Model D: *ssrA* copy number in hot water**						
Disinfectant	Chloramine − None	−1.69	0.26	−6.4	<0.01	<0.01
Intervention	Flushing − Shower	0.06	0.26	0.2	0.83	1.00
Pipe material	Aqua PE-Xa − Copper	0.47	0.26	1.8	0.07	0.22
Temp. set-point	60 °C − 49 °C	0.13	0.26	0.5	0.61	1.00

* Average marginal effects represent the mean difference in $\log_{10}$ reduction associated with a change in factor level (e.g., chloramine vs. no residual), averaged over the observed distribution of the remaining covariates.
† Avg. marginal effect ($\log_{10}$); positive values indicate greater immediate reductions associated with the first level listed in the contrast.
‡ Unadjusted *p* values provided for transparency; *p* values were adjusted for multiplicity within a model using the method of Holm[S9]

Table S10: Detection frequency of *Vermamoeba vermiformis* (amplification of 18S rRNA genes) in water samples before and after water use. Adapted with permission from Waak et al.[S2] Copyright 2024 The Water Research Foundation.

	**Before (stagnant)**			**After (fresh)**		
**Supply**	Positive	Total	% Positive	Positive	Total	% Positive
**No residual**						
Cold	13	24	54.2	0	12	0.0
Hot (49 °C)	3	12	25.0	0	6	0.0
Hot (60 °C)	12	12	100.0	0	6	0.0
**Chloramine**						
Cold	5	24	20.8	1	12	8.3
Hot (49 °C)	10	12	83.3	0	6	0.0
Hot (60 °C)	7	12	58.3	0	6	0.0

Table S11: Estimated model growth parameters based on 16S rRNA (total bacteria) and *ssrA* (*Legionella* spp.) gene concentrations in cold- and hot-water supplies. Adapted with permission from Waak et al.[S2] Copyright 2024 The Water Research Foundation.

			**Total bacteria**				***Legionella* spp.**			
**Supply**	**Parameter**	**Unit**	*n*	Median	Min.	Max.	*n*	Median	Min.	Max.
**No disinfectant residual**										
Cold	N_0	log[copies/L]	12	7.95	7.83	8.02	12	4.70	4.60	4.84
	N_{max}	log[copies/L]	12	8.87	8.62	8.97	10	6.28	5.72	7.38
	μ_{max}	1/day	12	1.15	0.44	4.01	11	0.39	0.04	1.21
	τ	days	12	0.61	0.17	1.58	11	1.80	0.57	15.32
	t_{max}	days	12	1.75	0.43	3.92	10	6.49	2.52	16.26
Hot (49 °C)	N_0	log[copies/L]	6	7.71	7.53	7.71	6	4.68	4.40	4.90
	N_{max}	log[copies/L]	6	8.84	8.79	9.15	2	6.23	6.00	6.46
	μ_{max}	1/day	6	3.36	1.60	8.00	6	0.11	0.07	0.57
	τ	days	6	0.23	0.09	0.43	6	6.76	1.22	9.24
	t_{max}	days	6	0.93	0.44	1.73	2	7.37	6.02	8.72
Hot (60 °C)	N_0	log[copies/L]	6	7.17	7.09	7.26	6	4.05	3.57	4.23
	N_{max}	log[copies/L]	6	8.83	8.60	8.90	2	6.27	5.94	6.61
	μ_{max}	1/day	6	1.04	0.81	5.07	6	0.11	0.06	0.36
	τ	days	6	0.67	0.14	0.86	6	6.52	1.93	10.89
	t_{max}	days	6	3.57	0.70	4.48	2	15.21	14.59	15.82
**Chloramine, 1 mg/L as Cl_2**										
Cold	N_0	log[copies/L]	12	7.90	7.78	8.09	Not modeled			
	N_{max}	log[copies/L]	7	8.88	8.82	9.42	(insufficient growth)			
	μ_{max}	1/day	12	0.61	0.12	4.45				
	τ	days	12	1.14	0.16	5.63				
	t_{max}	days	7	2.12	0.54	5.08				
Hot (49 °C)	N_0	log[copies/L]	6	7.37	6.49	7.46				
	N_{max}	log[copies/L]	6	8.93	8.83	9.11				
	μ_{max}	1/day	6	1.38	0.91	2.21				
	τ	days	6	0.50	0.31	0.76				
	t_{max}	days	6	2.54	1.95	4.42				
Hot (60 °C)	N_0	log[copies/L]	6	6.84	6.68	7.05				
	N_{max}	log[copies/L]	6	9.32	9.04	9.44				
	μ_{max}	1/day	6	1.39	1.22	2.73				
	τ	days	6	0.50	0.25	0.57				
	t_{max}	days	6	3.89	2.03	4.93				

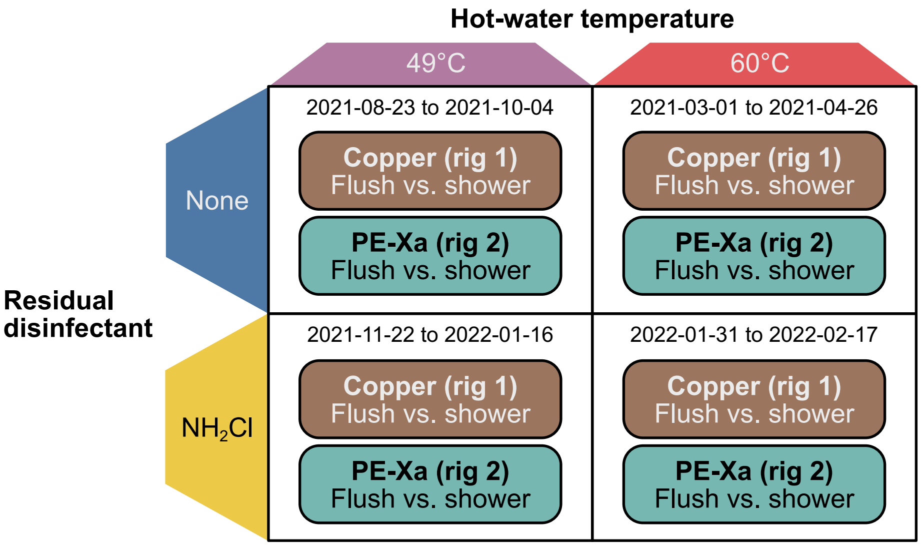

Figure S1: Factorial design of experiments in the pilot-scale building water system, with four temporal phases investigating combinations of hot-water temperature and residual disinfectant, plus the effects of flush versus showering (no flush) in copper versus PE-Xa pipes.

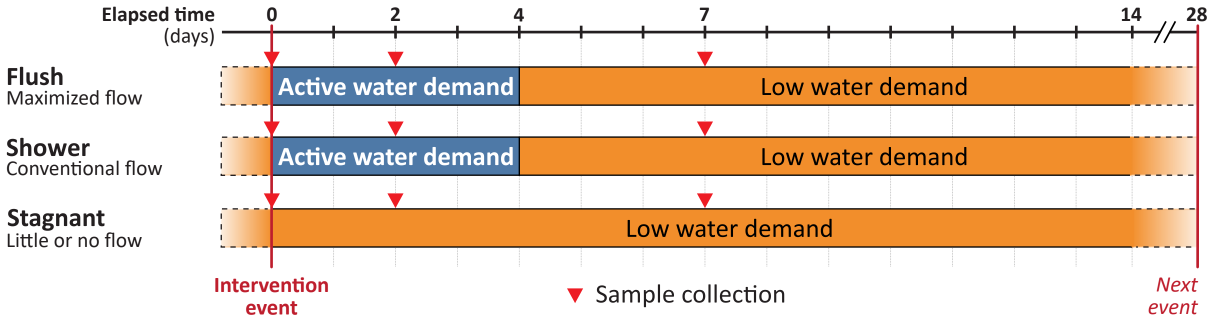

Figure S2: Experimental cycles: In each rig, one of three shower outlets was flushed after a period of low demand. The two non-flushed outlets served as controls, either resuming daily simulated showers or remaining stagnant. Water samples were collected as first-draws with a water age of at least 24 h. Adapted from Meegoda et al.[S10] Licensed under CC BY.

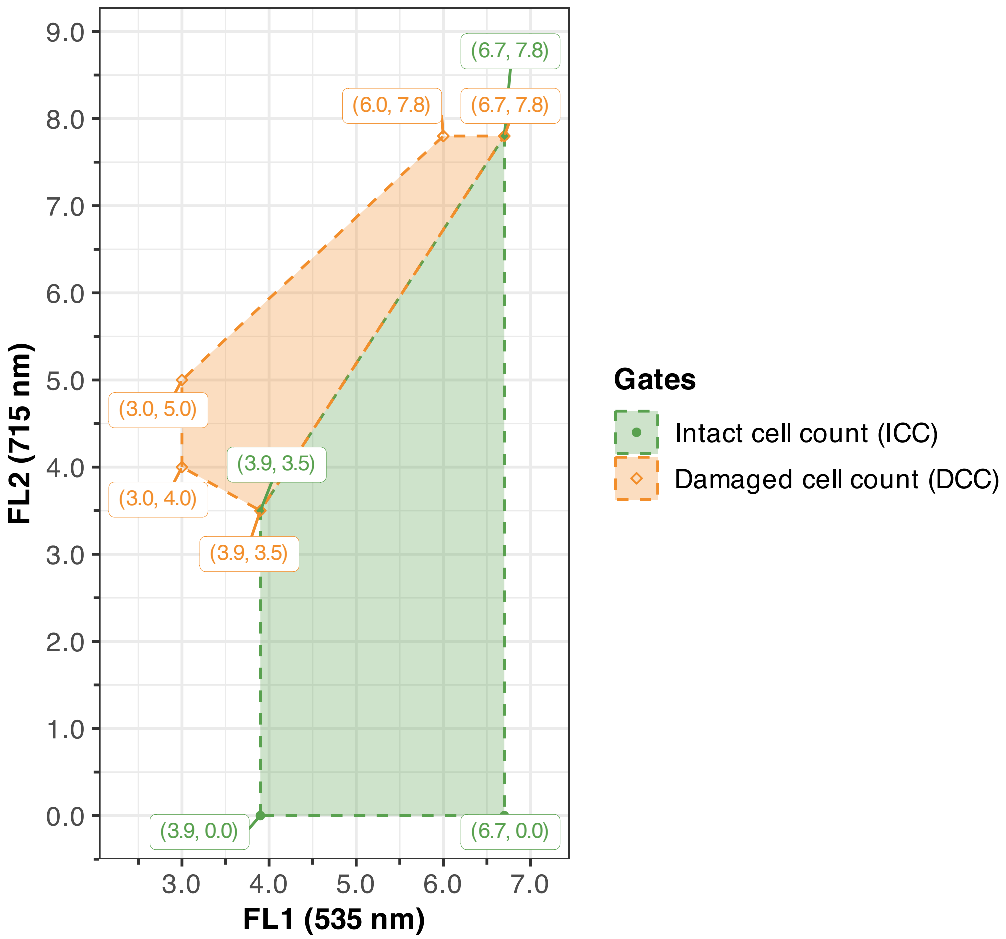

Figure S3: Flow cytometry gating strategy used to quantify total and intact cell counts. Electronic gates defined in green (FL1) and red (FL2) fluorescence space distinguish intact cell count (ICC) and damaged cell count (DCC); the sum of ICC and DCC constitutes the total cell count (TCC) for each sample. Gate boundaries and coordinates are shown.

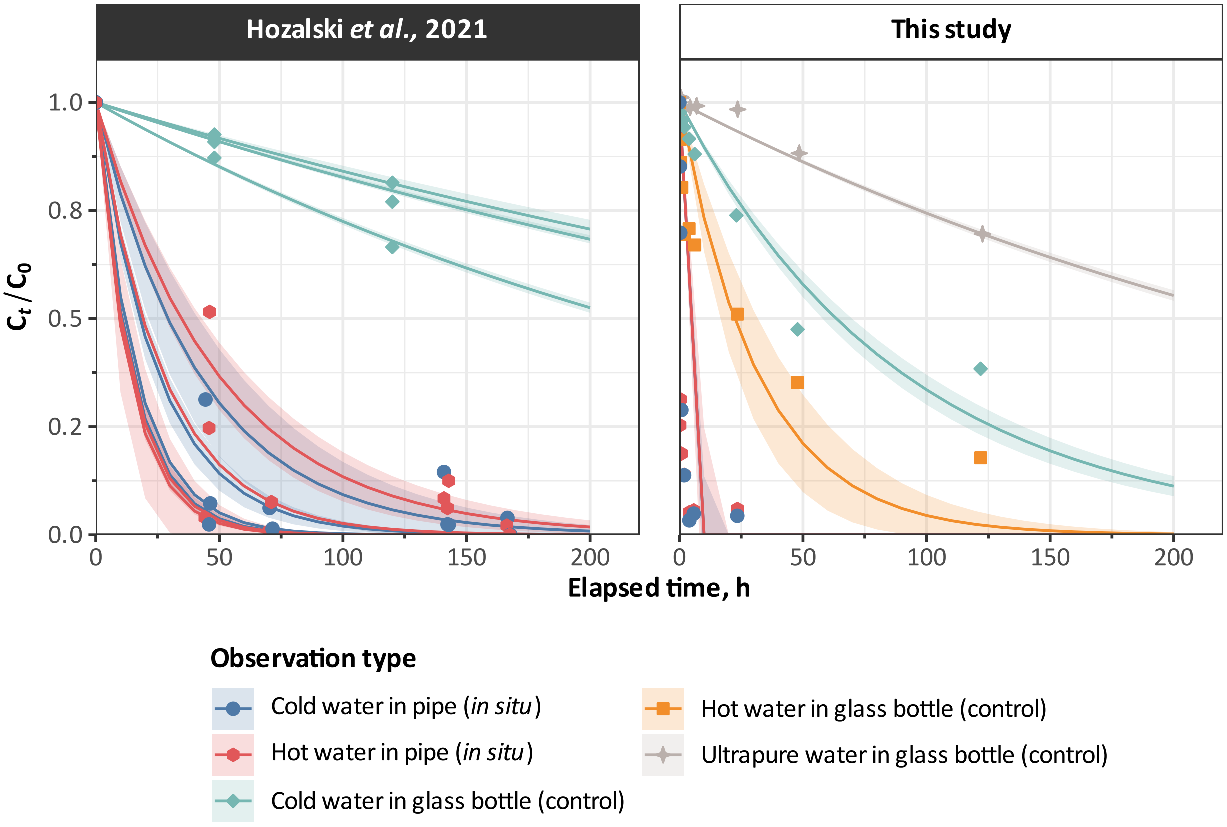

Figure S4: First-order decay of relative total chlorine concentrations in pipes and glass bottles over time in Hozalski et al.[S11] versus the present study. Ribbons indicate 95 % confidence interval. Adapted with permission from Waak et al.[S2] Copyright 2024 The Water Research Foundation.

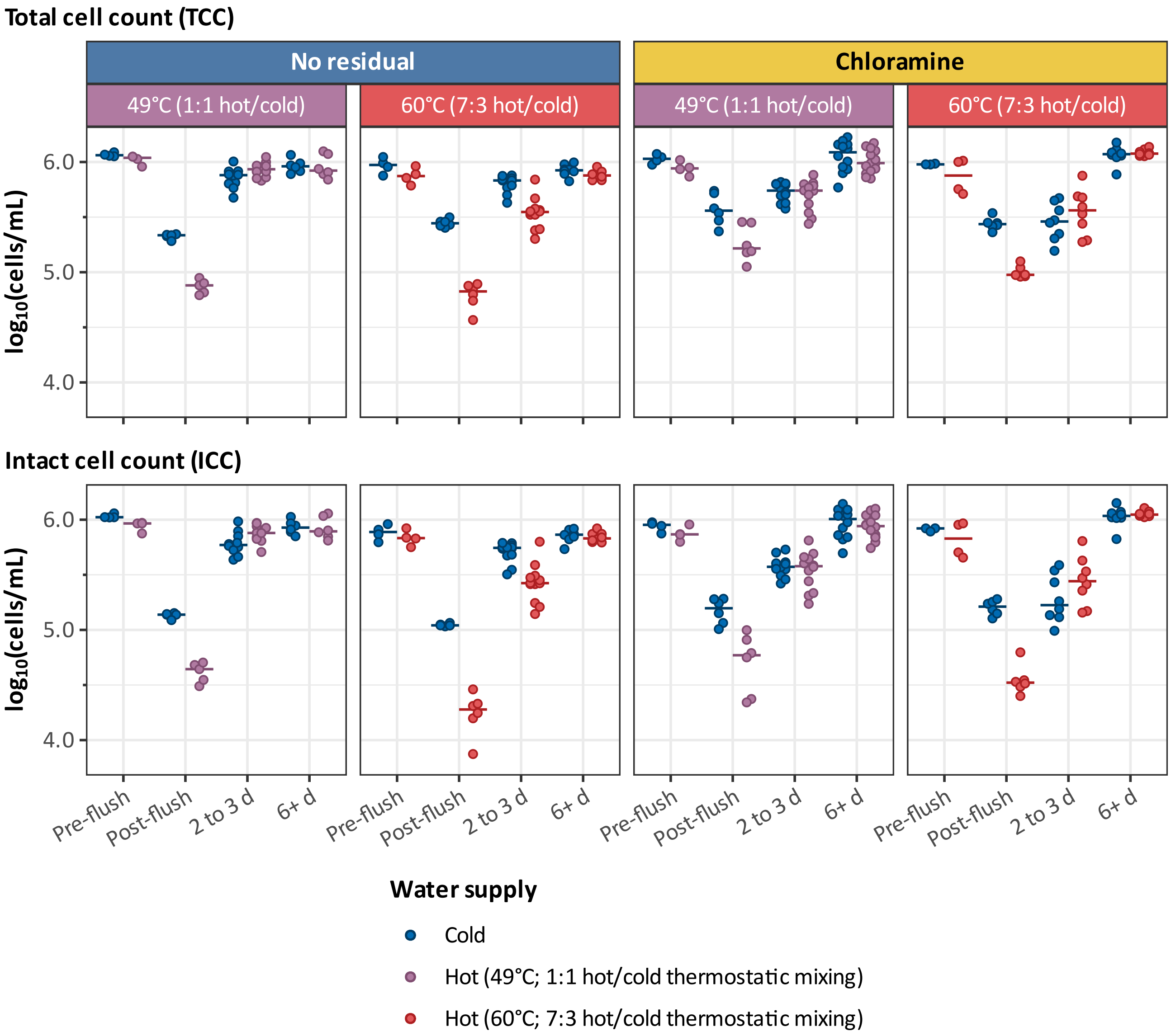

Figure S5: Monitoring of flow cytometric total and intact cell counts in water during pilot flushing experiments: pre- and post-flush and then monitoring during periods of no water demand at water ages of 2 to 3 days and 6 or more days.

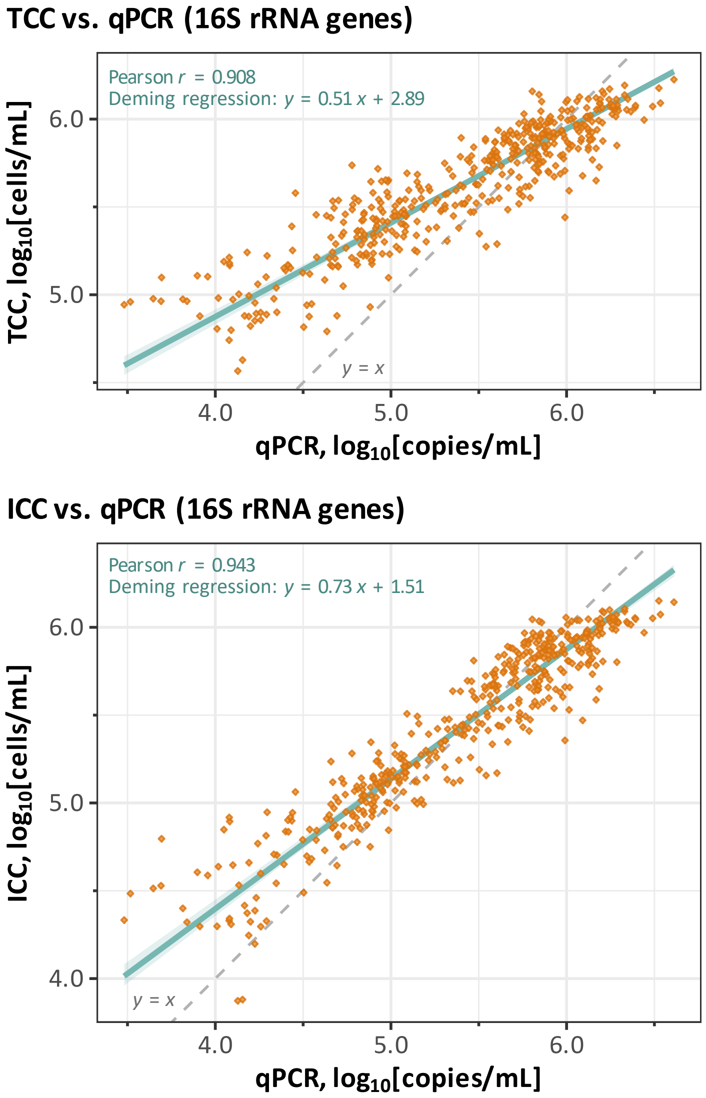

Figure S6: Total and intact cell counts (TCC and ICC) versus qPCR bacterial 16S rRNA gene concentrations among the water samples collected in this study (n = 456), with Pearson's product moment correlation coefficient and the Deming regression coefficients. The solid teal line is the regression with 95 % confidence interval, and the dashed grey line indicates equality ($y = x$).

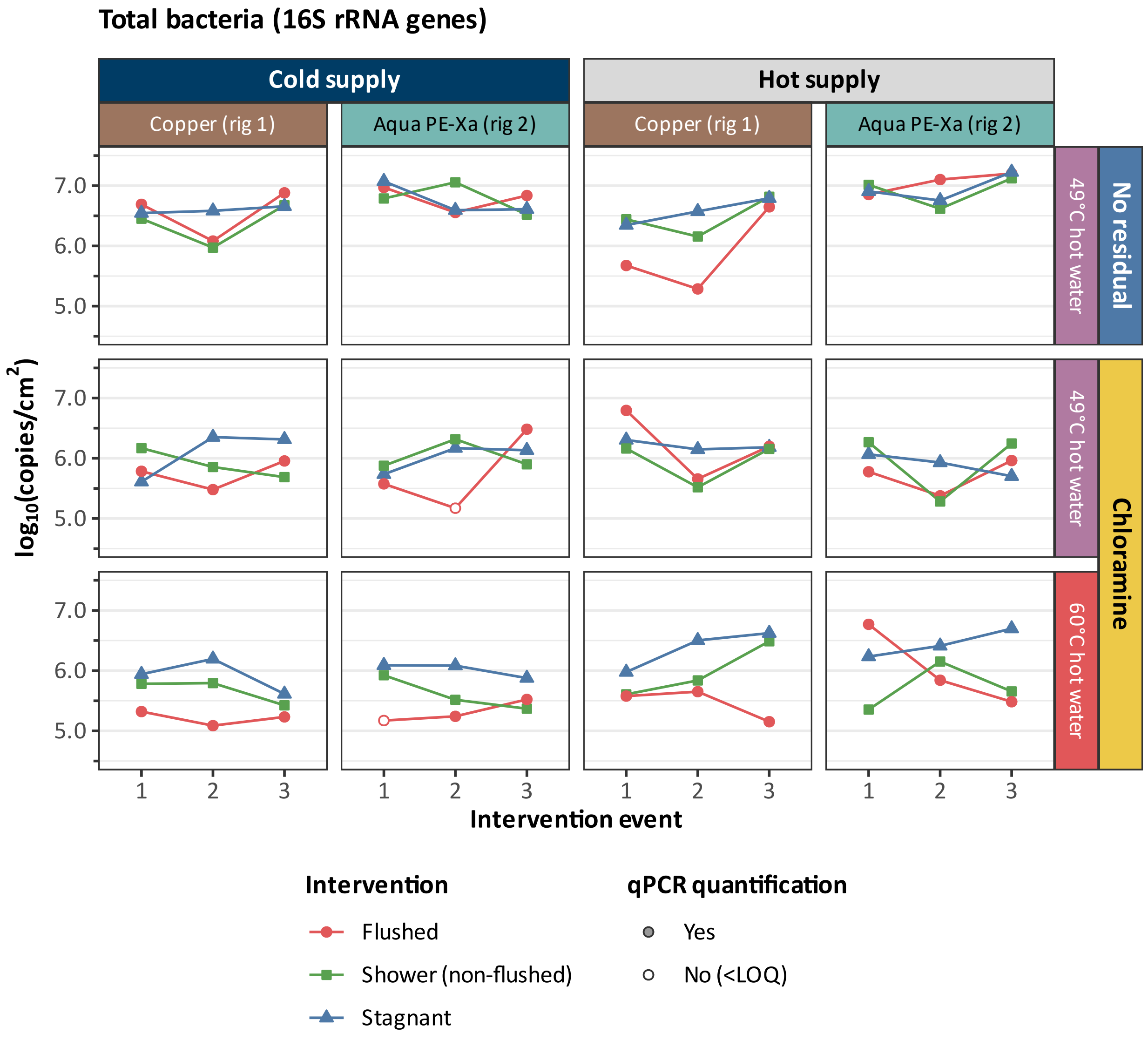

Figure S7: Total bacterial 16S rRNA genes in pipe biofilms. Samples without real-time quantitative polymerase chain reaction (qPCR) amplification or below the quantification limit are shown at the limit value. Biofilm samples from operation with no residual and hot-water temperature setpoint at 60 °C were excluded due to a different collection method. Adapted with permission from Waak et al.[S2] Copyright 2024 The Water Research Foundation.

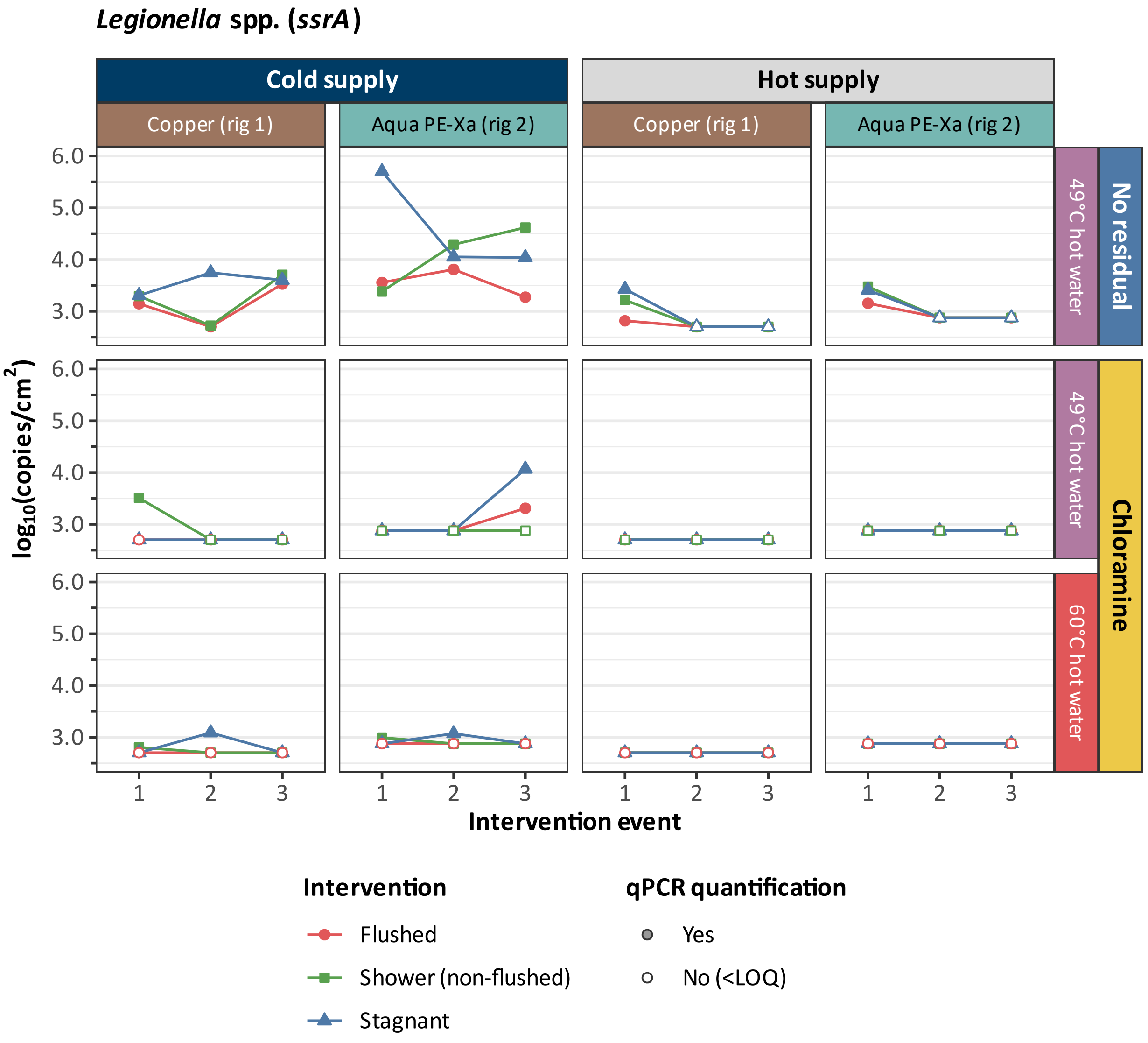

Figure S8: *Legionella* spp. *ssrA* in pipe biofilms. Samples without qPCR amplification or below the quantification limit are shown at the limit value. Biofilm samples from operation with no residual and hot-water temperature setpoint at 60 °C were excluded due to a different collection method. Adapted with permission from Waak et al.[S2] Copyright 2024 The Water Research Foundation.

References

(S1) Wagenmakers, E.-J.; Farrell, S. AIC model selection using Akaike weights. *Psychon. Bull. Rev.* **2004**, *11*, 192–196, DOI: 10.3758/BF03206482.

(S2) Waak, M. B.; Meegoda, C. S.; Hallé, C.; Hozalski, R. M. *Demonstrating the Effectiveness of Flushing for Reducing the Levels of* Legionella *in Service Lines and Premise Plumbing (Project No. 5033)*; The Water Research Foundation: Denver, Colorado, United States, 2024; https://www.waterrf.org/research/projects/demonstrating-effectiveness-flushing-reducing-levels-legionella-service-lines-and, accessed 2025-09-18.

(S3) Trondheim Municipality. Drikkevannskvalitet. 2020; https://www.trondheim.kommune.no/tema/veg-vann-og-avlop/vann-og-avlop/om-vann-og-avlop/drikkevannskvalitet, accessed 2021-03-03.

(S4) Waak, M. B.; LaPara, T. M.; Hallé, C.; Hozalski, R. M. Occurrence of *Legionella* spp. in water-main biofilms from two drinking water distribution systems. *Environ. Sci. Technol.* **2018**, *52*, 7630–7639, DOI: 10.1021/acs.est.8b01170.

(S5) Johansen, I. E. Assimilerbart organisk karbon i drikkevann i Trondheim. M.Sc. thesis, Norwegian University of Science and Technology, Trondheim, Norway, 2018.

(S6) Muyzer, G.; de Waal, E. C.; Uitterlinden, A. G. Profiling of complex microbial populations by denaturing gradient gel electrophoresis analysis of polymerase chain reaction-amplified genes coding for 16S rRNA. *Appl. Environ. Microbiol.* **1993**, *59*, 695–700, DOI: 10.1128/aem.59.3.695-700.1993.

(S7) Benitez, A. J.; Winchell, J. M. Clinical application of a multiplex real-time PCR assay for simultaneous detection of *Legionella* species, *Legionella pneumophila*, and *Legionella pneumophila* serogroup 1. *J. Clin. Microbiol.* **2013**, *51*, 348–351, DOI: 10.1128/JCM.02510-12.

(S8) Kuiper, M. W.; Valster, R. M.; Wullings, B. A.; Boonstra, H.; Smidt, H.; van der Kooij, D. Quantitative detection of the free-living amoeba *Hartmannella vermiformis* in surface water by using real-time PCR. *Appl. Environ. Microbiol.* **2006**, *72*, 5750–5756, DOI: 10.1128/aem.00085-06.

(S9) Holm, S. A simple sequentially rejective multiple test procedure. *Scand. J. Statist.* **1979**, *6*, 65–70.

(S10) Meegoda, C. S.; Waak, M. B.; Hozalski, R. M.; Kim, T.; Hallé, C. The benefits of flushing for mitigating *Legionella* spp. in non-chlorinated building plumbing systems. *Front. Water* **2023**, *5*, 1114795, DOI: 10.3389/frwa.2023.1114795.

(S11) Hozalski, R. M.; LaPara, T. M.; Zhao, X.; Kim, T.; Waak, M. B.; Burch, T.; McCarty, M. Flushing of stagnant premise water systems after the COVID-19 shutdown can reduce infection risk by *Legionella* and *Mycobacterium* spp. *Environ. Sci. Technol.* **2020**, *54*, 15914–15924, DOI: 10.1021/acs.est.0c06357.
